# Berberine Targets PKM2 to Activate the t-PA-Induced Fibrinolytic System and Improves Thrombosis

**DOI:** 10.3390/ph17091219

**Published:** 2024-09-17

**Authors:** Zeqi Sun, Tong Zhao, Xue Bai, Huimin Li, Jin Gao, Yutong Hao, Yiyang Li, Yanli Xie, Ange Hu, Qiang Huang, Xin Liu, Yong Zhang

**Affiliations:** 1 State Key Laboratory of Frigid Zone Cardiovascular Diseases (SKLFZCD), Department of Pharmacology, College of Pharmacy, Harbin Medical University, Harbin 150081, China; 2018173073@hrbmu.edu.cn (Z.S.); 202301161@hrbmu.edu.cn (T.Z.); 2022020416@hrbmu.edu.cn (H.L.); 2023020469@hrbmu.edu.cn (J.G.); 2023020443@hrbmu.edu.cn (Y.H.); 2023020404@hrbmu.edu.cn (Y.L.); 2023020484@hrbmu.edu.cn (Y.X.); 2023020422@hrbmu.edu.cn (A.H.); 2019173025@hrbmu.edu.cn (Q.H.); 2 State Key Laboratory—Province Key Laboratories of Biomedicine-Pharmaceutics of China, College of Pharmacy, Harbin Medical University, Harbin 150081, China; 3 Key Laboratory of Cardiovascular Research, Ministry of Education, College of Pharmacy, Harbin Medical University, Harbin 150081, China; 4 Research Unit of Noninfectious Chronic Diseases in Frigid Zone (2019RU070), Chinese Academy of Medical Sciences, Harbin 150081, China; 5 College of Pharmacy, Hainan University, Haikou 570228, China; xuebai@hainanu.edu.cn

**Keywords:** berberine, PKM2, t-PA, fibrinolytic system, thrombosis

## Abstract

Background: Arterial thrombosis, a condition in which thrombi form in arteries, can lead to various acute cardiovascular diseases and impact the quality of life and survival of patients. Berberine (BBR), a quaternary ammonium alkaloid, has been shown to treat these diseases. However, further exploration is needed to understand underlying mechanisms of BBR. Methods and results: Rats were administered BBR via intramuscular injection. Then, an FeCl_3_-coated filter paper was applied to a carotid artery to induce thrombosis. The size of the thrombus and the blood flow velocity were evaluated by carotid ultrasound. The shape of the thrombus was observed using staining and microscopy. The expression levels of mRNA and proteins were verified. Additionally, mass spectrometry and single-cell RNA sequencing analysis were conducted. The administration of BBR resulted in a significant reduction in the thrombus area and an extension of the thrombus-clogging time. Furthermore, BBR administration effectively reversed the decreasing tissue–plasminogen activator (t-PA) expression and alterations in fibrinolysis system of model group. Additionally, the expression of PKM2 was suppressed following BBR administration, and the overexpression of PKM2 inhibited t-PA expression. Conclusions: BBR ameliorates thrombosis by modulating expression of PKM2, subsequently impacting the expression of t-PA within fibrinolytic system. These preliminary findings suggest that BBR could be a potential preventive and therapeutic strategy for arterial thromboembolic diseases.

## 1. Introduction

Cardiovascular diseases (CVDs) continue to be the leading cause of mortality and disability globally [1]. Among these, thromboembolic diseases, which contribute to various acute cardiovascular events [2], have emerged as the predominant etiology of death-associated diseases [3,4]. Conditions such as myocardial infarction [5], ischemic stroke [6], heart failure [7], atrial fibrosis [8], and respiratory diseases [9] are closely associated with thrombosis, with over half of ischemic strokes attributed to arterial thrombosis [10,11,12]. The current clinical therapy is insufficient in mitigating the pathophysiological changes in arterial thromboembolic diseases, underscoring the critical need for innovative drug discovery in this area.

Thrombosis is initiated by the activation of the body’s blood-clotting mechanism, leading to the generation of fibrin. The fibrin that is produced, along with activated platelets, combines to create a thrombus or blood clot [13,14,15]. This process is related to two key systems: the anticoagulation system and the fibrinolytic system [16]. The fibrinolytic system, also referred to as the fibrinolysis system, holds particular significance [17]. Fibrinolysis is the process that involves the breakdown and liquefaction of fibrin, with fibrinolysin playing a pivotal role within the fibrinolytic system by degrading fibrin, thus preventing the formation of thrombi that could impede blood vessel function [18]. The activation of plasminogen and its subsequent conversion into fibrinolysin represent critical steps in the fibrinolytic process [19]. Central to the fibrinolytic system is tissue plasminogen activator (t-PA), a serine protease synthesized by vascular endothelial cells. T-PA is a specific activator of plasminogen within the fibrinolytic system, and its thrombolytic mechanism involves the activation of endogenous plasminogen to fibrinolysin [20,21,22]. T-PA has been extensively utilized in research on thrombolysis and thrombosis, including studies on acute myocardial infarction [23], intra-coronary thrombosis of ischemic stroke [24], and atherosclerosis [25], highlighting its significance in the field of arterial thrombosis [26,27]. Nevertheless, further investigation is warranted to explore the mechanisms by which t-PA regulates the fibrinolytic system in various thrombotic diseases, particularly arterial thrombosis.

Berberine (BBR), a benzyl quinoline alkaloid (C_20_H_18_NO_4_^+^) derived from plants such as Coptis chinensis Franch and Goldenseal, as well as other traditional Chinese medicines, exhibits stable quality and safety property in humans [28,29,30]. BBR has been shown to possess therapeutic effects in cardiovascular diseases, including the treatment of ischemic heart disease [31], heart failure [32,33], and atherosclerosis [34]. It could also decrease blood lipids [35] and blood sugar level [36,37]. Despite the well-established cardiovascular and thrombotic benefits of BBR, there remains a lack of detailed understanding regarding its specific mechanisms on thrombosis, with current research primarily focusing on gut-microbiota-associated studies [38]. Some research have demonstrated the efficacy and safety of BBR in peritoneal adhesion disease and diabetic cardiomyopathy [39,40]. Building upon these findings, it is important to investigate the efficacy and underlying mechanisms of BBR in thromboembolic diseases.

Pyruvate kinase (PK) is a crucial enzyme involved in the process of glycolysis. Pyruvate kinase isozyme type M2 (PKM2), predominantly presents in the liver, kidney, brain, and heart [41,42], plays a significant role in the regulation of cell growth [43,44]. PKM2 also exerts biological functions in glycolysis [45] and transcriptional regulation [46], with research primarily focusing on the process of tumor metabolism [47]. Nayak MK et al. have confirmed its role in platelet activation and arterial thrombosis [48,49]. Accordingly, PKM2 may play a contributory role in the pathogenesis of thromboembolic diseases and could serve as a potential target for novel drug therapies.

Hence, the aims of this study were as follows: (i) whether intramuscular injection of BBR can play a thrombolytic role through the fibrinolytic system; (ii) whether berberine can regulate PKM2 expression; (iii) whether PKM2 expression affects the thrombolytic function of t-PA and vascular fibrinolytic system.

## 2. Results

### 2.1. BBR Prevents Thrombosis in a Dosage-Dependent Manner

**Figure 1:**
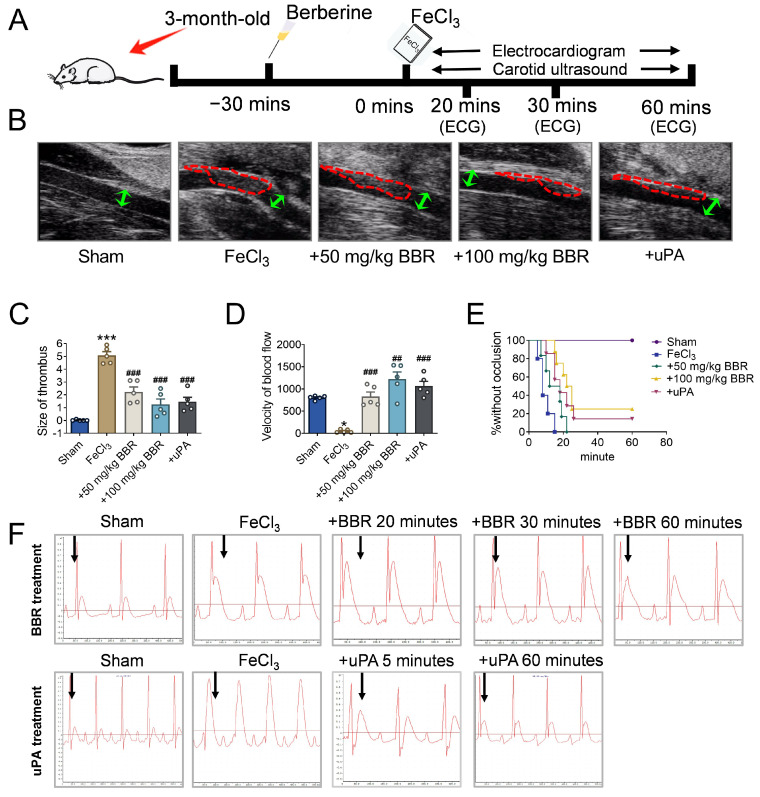
BBR prevents thrombosis in a dosage-dependent manner. (**A**) A time schematic diagram about construction of a common carotid artery thrombosis model and the administration of BBR in rats. (**B**) Doppler ultrasonography for detecting carotid thrombosis in rats. *n* = 5. The urokinase-type plasminogen activator (uPA) is a positive drug for blood clots. The red circles are the thrombus, and the green arrowheads show arterial width. (**C**) A comparison on the size of thrombus by ultrasonography in five groups. *n* = 5. (**D**) A comparison on the velocity of blood flow by ultrasonography in five groups. *n* = 5. (**E**) The percentage without occlusion in five groups. *n* = 5. (**F**) Electrocardiogram of rats in BBR treatment and uPA treatment. *n* = 5. * *p* < 0.05, *** *p* < 0.001 vs. sham. ^##^ *p* < 0.01 ^###^ *p* < 0.001 vs. FeCl_3_. Data were expressed by mean ± SEM.

To verify the effect of BBR on thrombosis, a common carotid artery thrombosis model was established according to Kurz method [50], and subsequent experiments were designed (Figure 1A). The study was conducted using SD rats, which were randomly divided into seven groups (*n* = 10 per group) and received intramuscular injections of BBR at doses of 100, 200, 400, 800, 1000, 2000, and 4000 mg/kg. The activities of the rats were continuously monitored, and the number of rat death within 72 h was recorded. The calculated LD50 was approximately 406.66 mg/kg. These findings suggest that intramuscular administration of BBR is a relatively safe delivery method, and the doses used in this study are well within the safety margin (Supplementary Figure S1A). The thrombus model was established after intramuscular injection with different concentrations (50 mg/kg and 100 mg/kg) of BBR, and the thrombus formation was observed during the following time. Doppler ultrasound diagnostic results showed that the arterial walls of rats in the sham operation group were smooth without thrombosis, while the rats treated with FeCl_3_ had obvious thrombosis, further indicating the successful establishment of the thrombus model (Figure 1B). Compared to FeCl_3_ treatment group (also called thrombus model group), the thrombus area in the artery inner wall was significantly reduced after the administration of BBR, and the reduction effect of 100 mg/kg BBR was significantly greater than 50 mg/kg (Figure 1B,C).

Moreover, compared with the sham operation group, the blood flow velocity of the thrombosis model group was significantly slowed down, while the blood flow velocity in the BBR treatment group gradually recovered, and the high concentration of BBR treatment could also significantly reduced the blood flow velocity compared to the low concentration group (Figure 1D). The results of arterial thrombosis occlusion illustrated that BBR could significantly prolong the thrombosis occlusion time and prevent the thrombosis occlusion caused by thrombosis (Figure 1E).

Meanwhile, we monitored the situation of arterial thrombus after the administration of BBR at different time points. With the administration of BBR, the thrombus blockage time was significantly extended from 20 min to 30 min compared with the thrombus model group (Supplementary Figure S2). We showed that the ST segment in the thrombus model group was significantly elevated, and this pathological change could be improved by BBR administration, and the improvement was more obvious at 60 min (Figure 1F). Collectively, these results indicated that the administration of BBR can obviously prevent the formation of thrombus, reduce the size of thrombus, and restore blood flow velocity.

### 2.2. BBR Regulates Fibrinolytic System Balance in Rat Model of Common Carotid Artery Thrombosis

**Figure 2:**
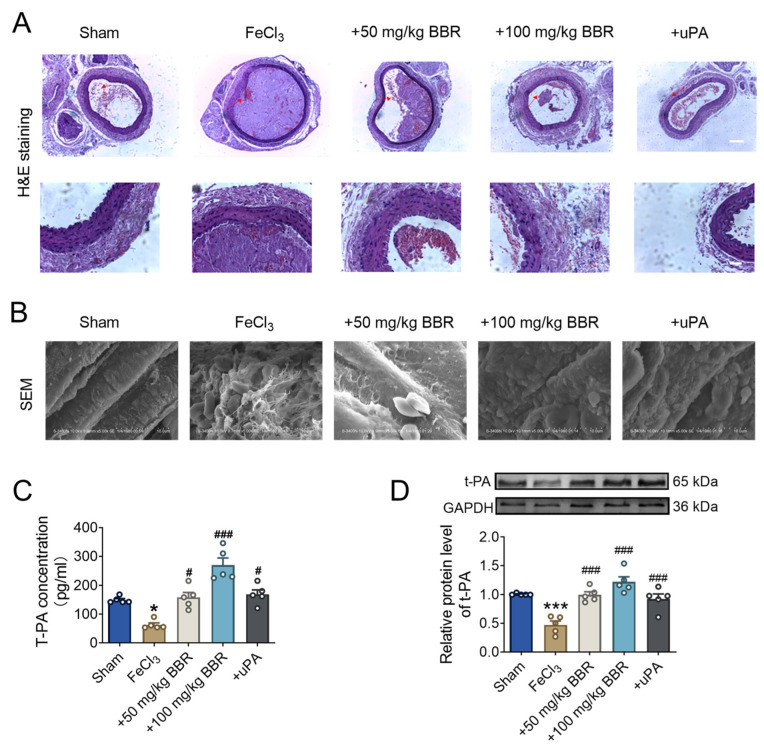
BBR regulates fibrinolytic system balance in rat model of common carotid artery thrombosis. (**A**) The H&E-stained image of the rat carotid artery was used to detect thrombus in five groups. *n* = 5. Scale bar = 200 μm 50 μm. Red arrows indicate thrombotic plates. (**B**) The scanning electron microscope was used for detecting carotid artery thrombosis in five groups. Scale bar = 10 μm. (**C**) Elisa kit that used to detect the plasma t-PA levels in five groups. *n* = 5. (**D**) The protein level of t-PA in carotid artery was detected using western blot. *n* = 5. * *p* < 0.05, *** *p* < 0.001 vs. sham. ^#^ *p* < 0.05, ^###^ *p* < 0.001 vs. FeCl_3_. Data were expressed by mean ± SEM.

To further validate the thrombus formation, we conducted histological analysis using H&E staining. Compared to the sham operation group, the arteries in the thrombus model group exhibited large and prominent thrombus. However, after treatment with BBR, the area of arterial thrombus was significantly reduced in a dose-dependent manner. (Figure 2A). SEM analysis revealed that the thrombus in the thrombus model group contained platelets, fibrin, and deformed red blood cells, which were not observed in the sham operation group. Notably, BBR treatment was able to effectively reduce thrombus formation at the animal level, with a more pronounced in the high-concentration BBR group (Figure 2B).

Fibrinolysis system is a key regulatory system for thrombosis. Therefore, we speculate whether BBR can promote thrombus dissolution by affecting the balance of fibrinolytic system. Elisa results showed that t-PA levels in the thrombus model group were significantly down-regulated compared with the sham operation group, indicating that the fibrinolytic system was significantly inhibited (Figure 2C). The reduction in the t-PA level could be reversed by the addition of BBR; the effect was more obvious in the high concentration group than low concentration group, respectively. Compared with the sham operation group, t-PA protein expression in the thrombus model group was significantly reduced, while BBR could gradually ameliorate the reduction in t-PA (Figure 2D), indicating BBR could improve the thrombosis caused by stimulation of FeCl_3_. Taken together, the above results indicated that BBR can regulate the fibrinolysis equilibrium system in the rat carotid artery thrombus model.

### 2.3. BBR Concentration and Incubation Time Affect t-PA Expression

**Figure 3:**
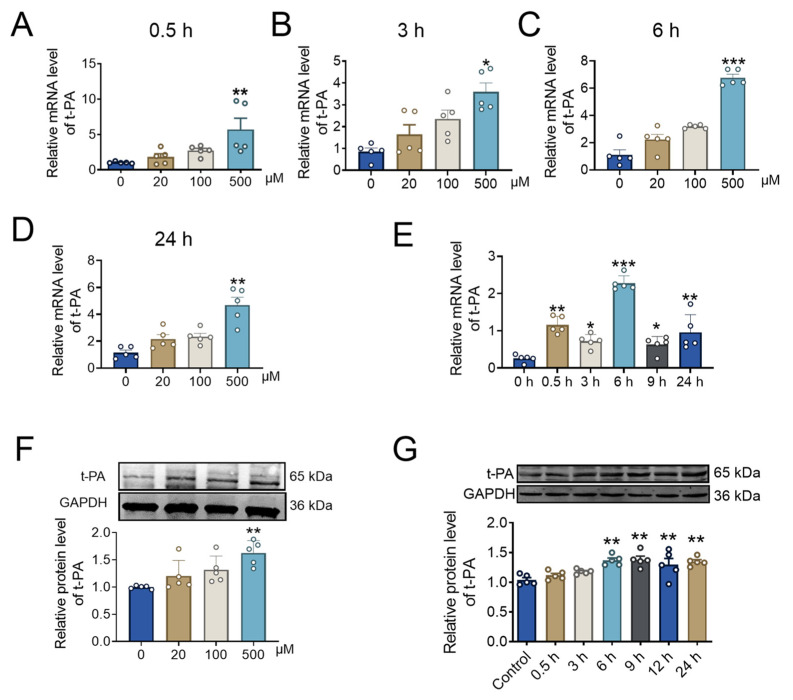
BBR concentration and incubation time affect t-PA expression. (**A**) The mRNA level of t-PA at 0.5 h of BBR treatment in different BBR concentration (0 μM, 20 μM, 100 μM, and 500 μM) by qRT-PCR. *n* = 5. (**B**) The mRNA level of t-PA at 3 h of BBR treatment in different BBR concentration (0 μM, 20 μM, 100 μM, and 500 μM) by qRT-PCR. *n* = 5. (**C**) The mRNA level of t-PA at 6 h of BBR treatment in different BBR concentrations (0 μM, 20 μM, 100 μM, and 500 μM) by qRT-PCR. *n* = 5. (**D**) The mRNA level of t-PA at 24 h of BBR treatment in different BBR concentration (0 μM, 20 μM, 100 μM, and 500 μM) by qRT-PCR. *n* = 5. (**E**) The mRNA level of t-PA at different times of BBR treatment in 500 μM BBR concentration by qRT-PCR. *n* = 5. (**F**) The protein level of t-PA at 6 h of BBR treatment in different BBR concentrations (0 μM, 20 μM, 100 μM, and 500 μM) by western blot. *n* = 5. (**G**) The protein level of t-PA at different times of BBR treatment in 500 μM BBR concentration by western blot. *n* = 5. * *p* < 0.05, ** *p* < 0.01, *** *p* < 0.001 vs. 0 μM, control or 0 h. Data were expressed as mean ± SEM.

The intima of the artery is composed of endothelial cells, which play an indispensable role in arterial thrombosis [51]. In order to further explore the influence of BBR on fibrinolysis system, different concentrations of BBR (0 μM, 20 μM, 100 μM, and 500 μM) with different incubation time were added to HUVECs. After 0.5 h of BBR treatment, compared with the control group (0 μM), the mRNA expression level of t-PA gradually increased following the increase in BBR concentration, and the expression level reached the peak at 500 μM (Figure 3A). 

Furthermore, we have also verified the expression level of t-PA under different concentrations of BBR at 3 h, 6 h, and 24 h. With the increase of BBR concentration, the mRNA expression level of t-PA gradually increased (Figure 3B–D). These results indicated that BBR could increase the mRNA expression level of t-PA in a dosage-dependent manner. Therefore, we next verified the effect of high concentration (500 μM) of BBR on t-PA at different times. The increased effect of t-PA by BBR became stronger over time, and the t-PA level reached the peak after 6 h of incubation (Figure 3E). With the increase in BBR concentration, the protein expression level of t-PA was significantly up-regulated, and 500 μM BBR had the most significant effect (Figure 3F). Moreover, the protein expression level of t-PA also increased with the incubation time, particularly after 6 h (Figure 3G).

We also determined the IC50 of BBR under a 6 h exposure in HUVECs. We treated the cells with various concentrations of BBR (0, 20, 100, 500, 750, 1000, and 1500 μM) and assessed cell viability using the CCK-8 assay. As shown in Supplementary Figure S1B,C, the IC50 was approximately 771.8 μM. Moreover, 500 μM of BBR had no obvious effects on the cell viability. These results indicated that the concentration of BBR used in our study did not adversely affect cell viability.

### 2.4. BBR Regulates PKM2 and Affects Thrombus Formation

**Figure 4:**
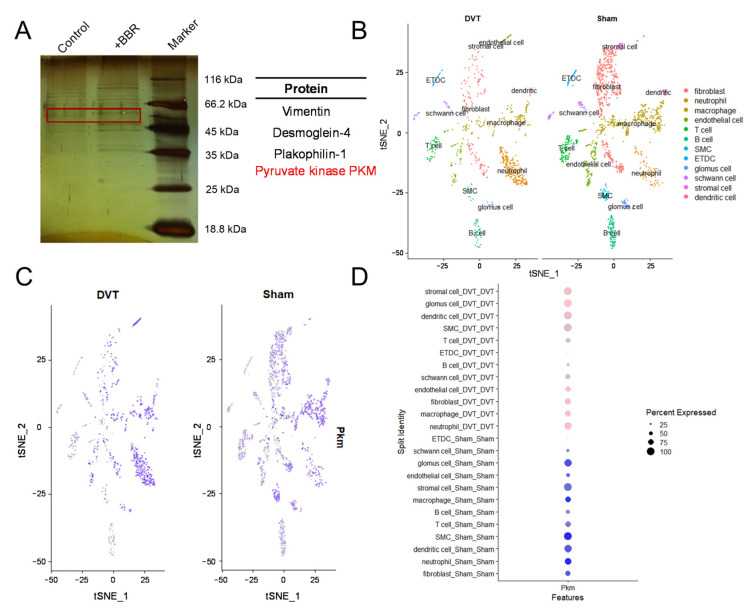
BBR regulates PKM2 and affects thrombus formation. (**A**) The mass spectrometry about BBR and its related protein. The red box represents PKM2. (**B**) UMAP plots of scRNA-sequencing data (GSE221978), which show the main cell types detected in DVT and sham groups. (**C**) FeaturePlot of PKM2 gene expression in all the cell types in DVT and sham groups. (**D**) DotPlot of PKM2 gene expression in all the cell types in DVT and sham groups.

Next, pull-down assays and mass spectrometry analysis on small molecules were undergone to explore the mechanism by which BBR affects thrombosis. In comparison to the control group, the BBR group exhibited a high-confidence PKM2-binding protein. (Figure 4A). It has been reported that the activator of PKM2 can promote PKM2 allostery, which would affect t-PA level and regulate fibrosis, thus affecting cirrhosis [52]. A recent study also found that allosteric PKM2 can be modulated to affect platelet activity and ultimately influence the process of arterial thrombosis [44]. Therefore, we speculated whether PKM2 was also involved in the regulation of the fibrinolysis system by BBR.

To verify the PKM2 gene expression level in the thrombus group and sham group, we analyzed a GEO single cell RNA-sequencing dataset GSE221978. UMAP showed 12 cell clusters (Figure 4B). Endothelial cells occupied 7.02% and 10.92% in the thrombus and sham group, respectively. The FeaturePlot analysis (Figure 4C) showed that the PKM2 gene was expressed in nearly all cell types in both the thrombus group and the sham group. Notably, the expression of PKM2 was significantly higher in the thrombotic endothelial cells compared to the sham group (Figure 4D, Supplementary Figure S3A). Furthermore, different up-regulated genes expressed in the thrombotic endothelial cells were used to undergo a GO enrichment analysis. The GO results showed that the different up-regulated genes expressed were mainly enriched in platelet-activation and fibrin-clot-related pathways (Supplementary Figure S3B). Overall, the above results further confirmed our hypothesis that BBR can be likely to influence the fibrinolysis system by regulating PKM2 and then have a positive effect on thrombolysis.

### 2.5. BBR Plays a Thrombolytic Role by Regulating the PKM2 and Then Affect t-PA

**Figure 5:**
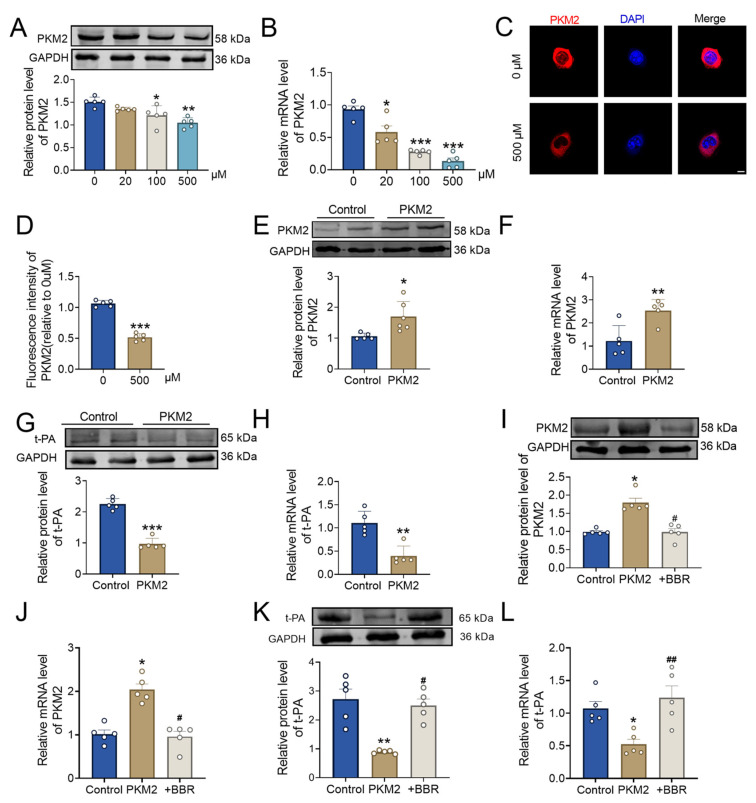
BBR plays a thrombolytic role by regulating the PKM2 and then affecting t-PA. (**A**) The protein level of PKM2 at 6 h of BBR treatment in different BBR concentrations (0 μM, 20 μM, 100 μM, and 500 μM) by western blot. *n* = 5. (**B**) The mRNA level of PKM2 at 6 h of BBR treatment in different BBR concentrations (0 μM, 20 μM, 100 μM, and 500 μM) by qPT-PCR. *n* = 5. (**C**,**D**) The localization and expression of PKM2 were detected via immunofluorescence assay. *n* = 5. Scale bar = 10 μm. (**E**) The protein level of PKM2 after overexpression of PKM2 using western blot. *n* = 5. (**F**) The mRNA level of PKM2 after overexpression of PKM2 via qPT-PCR. *n* = 5. (**G**) The protein level of t-PA after overexpression of PKM2 via western blot. *n* = 5. (**H**) The mRNA level of t-PA after overexpression of PKM2 via qPT-PCR. *n* = 5. (**I**) The protein level of PKM2 after overexpression of PKM2 and BBR treatment via western blot. *n* = 5. (**J**) The mRNA level of PKM2 after overexpression of PKM2 and BBR treatment via qPT-PCR. *n* = 5. (**K**) The protein level of t-PA after overexpression of PKM2 and BBR treatment via western blot. *n* = 5. (**L**) The mRNA level of t-PA after overexpression of PKM2 and BBR treatment via qPT-PCR. *n* = 5. * *p* < 0.05, ** *p* < 0.01, *** *p* < 0.001 vs. 0 μM or control. ^#^ *p* < 0.05, ^##^ *p* < 0.01 vs. PKM2. Data were expressed by mean ± SEM.

Then, we explored the relationship between PKM2 and t-PA after BBR treatment. With the administration of BBR, the protein expression level of PKM2 decreased significantly, and the PKM2 down-regulation was more obvious under the treatment of 500 μM BBR (Figure 5A). The PKM2 mRNA expression level was significantly decreased after BBR treatment, and the effect of high concentration was more obvious (Figure 5B). Immunofluorescence results demonstrated that, compared with the control group, the fluorescence level of PKM2 was significantly reduced after BBR administration (Figure 5C,D). 

At the cellular level, transfection with a PKM2 overexpression plasmid showed that the mRNA and protein levels of PKM2 were significantly higher in the overexpression group compared to the control group, confirming successful transfection (Figure 5E,F). Subsequently, the protein expression level of t-PA was significantly downregulated in the PKM2 overexpression group compared to the control group (Figure 5G). The mRNA level of t-PA was downregulated following PKM2 overexpression (Figure 5H). We then further verified the effect of BBR on this regulation. The expression changes in PKM2 and t-PA caused by PKM2 overexpression could be reversed upon the addition of 500 μM BBR (Figure 5I–L). Collectively, these results indicated that PKM2 can significantly inhibit the expression of t-PA and affect its function. Additionally, PKM2 can play a thrombolytic role by binding to t-PA. 

## 3. Discussion

Thromboembolic diseases, particularly arterial thrombosis, present a significant threat to human health and survival [2]. For these diseases, reducing thrombosis, thrombolytic techniques, and drug delivery remain considerable challenges. Consequently, there is an urgent need to identify safe and effective therapies to advance arterial thrombosis thrombolysis technology. BBR, a naturally occurring compound extracted from plants like Coptis chinensis Franch and Phellodendri Chinensis Cortex, is among the potent active ingredients found in Coptis and other botanical remedies. BBR possesses diverse pharmacological effects, encompassing anti-inflammatory properties, antimicrobial activities, anticancer potential, regulation of lipid metabolism, hypoglycemic effects, and immunomodulation [53,54]. Recently, BBR has gained recognition in the realm of cardiovascular diseases, particularly for its potential in managing conditions such as hypertension, hyperlipidemia, insulin resistance, arrhythmia, and platelet aggregation [55,56]. Our experimental results showed that BBR plays a therapeutic role in thrombosis, reduces the size of thrombus area and delays the time of arterial thrombosis. To a certain extent, BBR targets PKM2, which enhances t-PA expression within the fibrinolytic system, thereby promoting thrombolysis. Our study preliminarily offers a potential safe and effective strategy for the treatment of thrombosis.

Although current researches have indicated a strong correlation between BBR and thrombotic events, the precise mechanism underlying this association remains inadequately explored. Therefore, elucidation of the exact mechanism by which BBR promotes thrombolysis will provide potential strategies for the prevention and treatment of thrombotic diseases. Consistent with previous findings of BBR in the treatment of thrombus, that is the occurrence and percentage of thrombus or positive platelets were observed after injection of sodium laurate or intraperitoneal injection of carrageenan to assess and confirm the formation of thrombi [57,58]. Our data also corroborated the above clinical manifestations of thrombosis. Notably, the distinction lies in our direct induction of thrombosis through carotid artery infiltration with FeCl3 solution [50]. This method resulted in pathological changes reflective of those observed in clinical patients, facilitating a more direct examination of the impact of drugs on thrombosis. Furthermore, current research on thrombosis treatment predominantly focuses on oral administration methods, such as the study by Zhang HJ et al., which investigated BBR’s thrombosis-preventing effects through the promotion of phenylacetic acid degradation, following a 14-day administration period [38], and subsequent effects were further explored using comprehensive metabolomics and molecular docking approaches. However, BBR exhibits low oral absorption and bioavailability in both animal models and humans [59]. Askri H reported that intramuscular injection of BBR and chlorpromazine could improve intestinal over secretion induced by E. coli heat-unstable enterotoxin, which proved that intramuscular injection could promote the absorption of BBR by tissue cells to enhance the therapeutic effect [60]. Different routes of administration lead to differences in bioavailability and rate of drug absorption, ultimately resulting in different LD50 values. The LD50 for berberine was reported to be 9.04 mg/kg and 57.61 mg/kg for intravenous (IV) and intraperitoneal (IP) administration, respectively [61]. Intramuscular injection (IM) is considered to be safe, as supported by our experiment, which monitored the physiological condition of rat’s post-injection and calculated their LD50 to be 406.6663 mg/kg. Therefore, we confirmed the impact of BBR on thrombotic diseases through intramuscular injection, underscoring its potential therapeutic benefits.

Most studies on the relationship between BBR and thrombotic diseases have focused on altering the composition of intestinal microbiota and related metabolites to inhibit thrombosis, that is, BBR has been shown to inhibit thrombosis by either reducing the production of trimethylamine N-oxide (TMAO) or promoting the degradation of phenylacetic acid [62]. Additionally, it can regulate vitamin-K-catalyzed circulation to inhibit thrombosis, with a duration of action observed to be six weeks [63]. Zhang et al. discovered that BBR can inhibit ADP-induced platelet activation and decrease the expression levels of inflammatory factors such as TNF-α, IL-1β, ICAM-1, and RANTES in serum and heart tissue when orally administered for 14 days, thus exhibiting potential in ameliorating thrombosis [58]. These findings suggested that the mechanism of BBR affecting thrombus was complex and associated with the fibrinolytic system. In our experiment, intramuscular injection of BBR 30 min before the induction of thrombosis resulted in a significant reduction in the area of thrombus and the best effect was achieved one hour after administration. As scientists deepen their understanding of platelet function, the process of thrombosis becomes clearer. Thrombus, consisting of insoluble fibrin and platelets, form when the balance between the coagulation system and the fibrinolytic system is disrupted. Fibrinolysis in the bloodstream is primarily driven by t-PA, which converts plasminogen (PLG) into plasmin (PL), subsequently degrading fibrin to maintain vascular patency [64]. The fibrinolytic system has been proven to be beneficial in thrombosis treatment. The activity of t-PA is swiftly inhibited by plasminogen activator inhibitors, vital components of the fibrinolytic system acting as “scavengers” in blood vessels. Here, we confirmed that BBR aided in restoring t-PA levels in the fibrinolytic system, consistent with recent studies indicating that inhibiting NDRG1 significantly reduced t-PA expression, affecting thrombotic response and vascular remodeling [65]. Moreover, we demonstrated that the expression of t-PA was significantly reduced in the FeCl_3_-induced thrombosis group, while the expression level of t-PA was significantly restored after the application of BBR. These observations clearly indicate that intramuscular injection of BBR heightens t-PA expression in the fibrinolytic system, facilitating thrombus dissolution. The duration and concentration of a drug’s action are crucial in the treatment of almost all diseases. In this study, we tested the effects of BBR drugs with different concentrations and different action times on t-PA. The results indicated that high-concentration BBR with a 6 h action period had the most pronounced impact on t-PA within the fibrinolytic system. This finding underscores a novel aspect of BBR’s pharmacological action.

The development of biological or chemical agents targeting thrombosis highlights the potential of arterial endothelial cells as a promising therapeutic focus [66]. BBR has been identified as a novel ACSL4 inhibitor with the ability to inhibit endothelial ferroptosis and atherosclerosis [67]. Consequently, understanding the pivotal role of endothelial cells in cardiovascular diseases represents a core strategy for effective treatment. Currently, endothelial cells are being utilized as a therapeutic target in thrombotic disease management, with clinical translational studies investigating their impact on stent restenosis and thrombosis [68], in which the observation of the heterogeneous distribution of coagulation and fibrinolytic factors in the endothelia and the adaptation of the endothelia to various stimuli is also helpful for the diagnosis and management of bleeding and thrombotic complications [69]. Therefore, comprehending the involvement of endothelial cells in thrombus treatment holds paramount significance.

Pyruvate kinase (PK) is a crucial enzyme in glycolysis, catalyzing the final step of the glycolysis process by transferring phosphate groups from phosphoenolpyruvate (PEP) to ADP and the subsequent pyruvate and ATP production [70]. The M2 isoform of pyruvate kinase, PKM2, exhibits diverse effects, particularly in cardiac and vascular studies. Research has indicated that the hydroxylation of PKM2 by Jmjd4 can promote the degradation of PKM2 in cardiomyocytes and prevent dilated cardiomyopathy [71]. Additionally, targeting the PARP1–PKM2 axis has shown promise in treating right ventricular failure linked to pulmonary hypertension [40]. Studies in the arteries have shown that the loss of myeloid PKM2 can enhance pinocytosis and reduce atherosclerosis [72]. Recent investigations by Nayak MK et al. have unveiled the role of PKM2 in regulating PI3K-mediated Akt- and GSK3-signaling pathways, influencing platelet function and arterial thrombosis [48]. Salvianolic acid A has been demonstrated to modulate endothelial cell pyroptosis by directly targeting PKM2 to ameliorate diabetic atherosclerosis [73]. Furthermore, research has indicated that PKM2 plays a crucial role in regulating endothelial cell junction dynamics, collective migration, and angiogenesis in in vitro and in vivo settings [74]. Therefore, the effect of PKM2 on endothelium is worth studying in detail. In this study, we used single-cell-RNA-sequencing data (GSE 221978) analysis to explore the expression of PKM2 in each cell of the DVT animal model. Our analysis results illustrated that PKM2 expression was significantly elevated in the DVT animal model compared to the sham group. Further analysis of the cell subsets showed that the changes in endothelial cells were also consistent. Therefore, we then used endothelial cells to verify the relationship between PKM2 and t-PA. Through a small molecule pull-down experiment and mass spectrometry analysis, the interaction of BBR with PKM2 in endothelial cells was established, suggesting a probable regulatory role of BBR on arterial thrombosis dissolution via PKM2 modulation. We speculate whether PKM2 is related to the fibrinolytic system regulated by BBR. The results show that BBR is involved in the regulation of PKM2 and has a key effect on t-PA with the fibrinolytic system in endothelium cells and ultimately plays a role in thrombolysis. This study expands the understanding of the mechanisms underlying BBR’s regulation of thrombosis, while also offering novel insights into endothelial metabolism and function.

This study has several limitations. First, the use of ultrasound in this study may introduce some variability due to potential differences in ultrasound transducer positioning around the artery across different animals [75]. To validate our findings more accurately, future studies could incorporate multiple in vivo imaging technologies such as OCT and angiography. Second, the small sample size and the absence of female rats in the animal experiments may contribute to some variability in the results. A larger sample size including both male and female rats will be used in future studies to further validate the reliability and generalizability of our conclusions. Finally, while we identified a potential regulatory relationship between PKM2 and t-PA, further investigation is needed to fully elucidate the underlying mechanisms of this interaction.

In summary, our study demonstrates that intramuscular injection of BBR can induce a thrombolytic effect by modulating the equilibrium of the fibrinolytic system. Specifically, our research indicates that BBR regulates the expression and functionality of PKM2 in both arteries and endothelial cells, which in turn influenced t-PA within the fibrinolytic cascade. These findings provide a new potential therapeutic target and strategy for the clinical management of thrombus-related disorders. Further investigations are needed to elucidate the exact molecular mechanisms governing the interplay between PKM2 and the fibrinolytic system, as well as to evaluate the translational potential of BBR for treating thrombotic diseases.

## 4. Materials and Methods

### 4.1. Animals

The Sprague–Dawley (SD) rats used in this study were procured from Changsheng Bio-technology Co., Ltd. (Lianyungang, China). All animals involved in the experiment were housed in a standard environment, with a temperature of 23 ± 1 °C, humidity of 55 ± 5%, and ad libitum access to food and water. The experimental protocol in this study and the experimental process were conducted in accordance with the Guide for the Care and Use of Laboratory Animals (NIH publication, 1996) and approved by the Ethics Committee of Harbin Medical University with approval No. IRB3075724.

### 4.2. FeCl_3_-Injury-Induced Carotid Thrombosis

After the rats were acclimated to the environment, the rats were injected intraperitoneal with tribromoethanol (0.2 g/kg, Harbin Pharmaceutical Group Co., Ltd., Harbin, China) to become anesthetized, and then the common carotid artery thrombosis model was modified according to the Kurz method [50]. The neck skin was prepared, followed by the application of iodine tincture and deionization using 75% ethanol on the skin surface. Sterilization equipment was utilized to separate the neck skin along the midline, and a glass-dissecting tool was then used to isolate approximately 3 cm segments of the common carotid arteries on both sides. To protect the perivascular tissues, a plastic wrap cling film was placed between the common carotid artery and the surrounding tissues. After the preparation steps, a small piece of filter paper (0.1 cm × 1 cm) containing 35% FeC1_3_ solution (Sigma, 12322, St. Louis, MO, USA), 20 μL, was applied to the left common carotid artery, and the filter paper was removed 30 min after application. BBR was administered via intramuscular injection 30 min prior to the procedure, at different concentrations (50 mg/kg and 100 mg/kg). In the sham operation group (normal control group), all surgical procedures were replicated from the model group, with the exclusion of FeCl_3_ solution, utilizing normal saline instead. The final number of rats in each group and each experiment was 5. 

### 4.3. Cell Viability

HUVECs were plated in 96-well cell culture plates. Twenty-four hours later, the medium was removed and replaced with different concentrations of berberine (0, 2, 100, 500, 750, 1000, and 1500 μM). After 6 h, the cell viability was determined by using Cell Counting Kit-8 (CCK8, abbkine, Wuhan, China) following the manufacturer’s instructions. The absorbance of the converted dye was measured at the wavelength of 450 nm, and the absorbance was directly proportional to the cell viability.

### 4.4. Acute Toxicity

The rats were randomly divided into five groups, and the same volume of berberine was injected intramuscularly. The concentrations in the seven groups (*n* = 10) were 100, 200, 400, 800, 1000, 2000, and 4000 mg/kg, respectively. The activities of the rats were continuously monitored, and the number of rat death within 72 h was recorded. Then, the LD50 of the rats was calculated and obtained.

### 4.5. Doppler Ultrasound Diagnostic Apparatus

Carotid thrombosis was detected in the rats after BBR administration and FeCl_3_ solution induction modeling. After abdominal anesthesia, the rats were secured on an ultrasound table with adhesive tape. Subsequently, a small animal Doppler ultrasound system (VisualSonics, Toronto, ON, Canada) imaging system was employed to detect carotid thrombosis. The instrumental analysis software Doppler (Vevo 2100) flow imaging was utilized to analyze the vascular status, including calculation of blood flow (velocity of blood flow), size of thrombus, and arterial occlusion (%without occlusion).

### 4.6. Electrocardiogram (ECG)

The ECG was assessed using the BL420s biosignal acquisition system. Following anesthesia, the SD rats were positioned supine on the operating table, with needles inserted into the subcutaneous regions of their upper limbs and right lower limbs. Electrocardiogram electrodes were securely affixed to the upper limbs and right lower limbs of the rats. The lead ECG was then recorded using the BL420S biosignal acquisition system. ST elevation measurements were taken to evaluate thrombosis and ischemic events.

### 4.7. Scanning Electron Microscopy (SEM)

The detailed morphological structure was observed via a scanning electron microscope (Hitachi S-3400N, Tokyo, Japan). Blood smears were prepared by placing the thrombus tissues from the rats in various treatment groups onto slides and fixing them with paraformaldehyde for 2 h. After dehydration with gradient alcohol, observations were made under the scanning electron microscope.

### 4.8. Enzyme-Linked Immunosorbent Assay (ELISA) Kit

ELISA (Abcam, ab198510, Cambridge, MA, USA) kit was specifically designed to quantitatively measure the levels of active t-PA. The plates were pre-coated with antibodies. The plasma was added to the appropriate wells, and the primary antibody was added to detect the antibody at room temperature. Each well was then washed to remove any unbound substances. Subsequently, an HRP (Horseradish Peroxidase)-conjugated secondary antibody was added to bind with the primary antibody. The plates were incubated at room temperature, followed by the addition of the developer solution until color development occurred. The stop solution was added to halt the color reaction. The color development was recorded immediately after the stop solution was added.

### 4.9. Hematoxylin and Eosin (H&E) Staining

The tissues were cleaned with normal saline and fixed with 4% polyformaldehyde (Biosharp, Hefei, China) at room temperature. After dehydration, the tissues were embedded with paraffin wax. The paraffin sections were adjusted to be 5 μm thick. Next, the slices were put into 95% ethanol twice, for 3 min each time, stained with hematoxylin in a kit (Biosharp, Hefei, China) for 45 s, differentiated by 1% hydrochloric ethanol for 30 s, and rinsed under running water for 10 min. Next, the eosin in the kit was stained for 2 min; 80% ethanol, 95% ethanol, and anhydrous ethanol were dehydrated for 1 min each; and xylene was transparent for 2 min. The paraffin sections were sealed with neutral resin and dried overnight in a constant temperature oven at 56 °C. Pathological changes were observed and recorded under light microscope (Olympus, Tokyo, Japan).

### 4.10. Pulldown and Mass Spectrometry

The pulldown test (genecreate.cn) was used to detect proteins interacting with BBR. Firstly, the human umbilical vein endothelial cells (HUVECs, obtained from Wuhan Pricella Biotechnology Co., Ltd., Wuhan, China) used in the experiment were cultured, and cell lysates (1 × 10^7^) were obtained after treatment. First, biotin was connected to BBR for further pulldown. The hydroxyl group was modified by modifying the berberine 9-position methoxy structure, the carboxyl group of biotins was activated to acylchloride. Reaction activity was improved and the target compound was successfully synthesized (9-0-Biotin Tetrahydropalmatine Ester). NMR and IR were used to confirm its structure. The pulldown experiment was performed by connecting the 9-0-Biotin Tetrahydropalmatine Ester with the magnetic beads coated with avidin. The proteins interacting with BBR were captured by the avidin on magnetic beads in the total protein lysates from HUVECs. The proteins were electrophoreted with SDS-PAGE and silver-dyed to observe the pulled-down protein. Protein identification was performed by LC/MS mass spectrometry. Following retrieval and analysis by Proteinpilot software (5.0.2), proteins with a confidence level of more than 95% and at least one unique peptide segment were included in the analysis. Commonly contaminated proteins such as keratin, antibody protein, and serum albumin were eliminated. Finally, the proteins identified in both the control and experimental groups were determined.

### 4.11. Cell Culture and Transfection

In this experiment, HUVECs were used as experimental cells for exploration. HUVECs were transfected with the PKM2 overexpression plasmid (Integrated Biotech Solutions, IBSBIO, Shanghai, China); the corresponding volumes of Opti-MEM (Gibco, Grand Island, NY, USA) and X-treme Reagent (Roche, Basle, Switzerland) were added to one tube, and the corresponding volumes of Opti-MEM and PKM2 overexpression plasmid were added to the other tube, mixed, and added to the serum-free medium. After 6–8 h, the medium was changed to the medium with the serum. The cells were cultured for 48 h and then treated. BBR was added to the cells at different times after transfection.

### 4.12. Immunofluorescence

HUVECs were inoculated on 24-well glass slides and cultured for 12 h. The cells were pretreated with BBR (500 μM) for 6 h. The cells were washed with PBS and fixed with 4% paraformaldehyde at room temperature for 30 min. The cells then penetrated PBS containing 1% BSA (Roche) and 0.1% TritonX-100 (Sigma, St. Louis, MO, USA) for 30 min. Then, the cells were sealed with goat serum (Beijing Zhong Shan-Golden Bridge Biological Technology Co., Ltd., Beijing, China) for 1 h and washed once with PBS. Finally, the cells were incubated with the specific primary antibody at 4 °C overnight, and then a goat anti-rabbit 594 secondary antibody (Invitrogen, Carlsbad, CA, USA) was used at room temperature for 1 h away from light. The cells were immobilized with DAPI-containing anti-fluorescence quencher and slide for 20 min after cleaning. The fluorescence signal was analyzed by fluorescence microscopy.

### 4.13. Western Blot

HUVECs were lysed with a RIPA buffer containing a protease inhibitor (Solarbio Science&Technology Co., Ltd., Beijing, China). Artery and HUVECs samples (70 µg) were separated by PAGE gel (150 V) electrophoresis (BIO-RAD, Hercules, CA, USA) for 60 min. The gel was then successively transferred to the nitrocellulose membrane (PALL), and the parameter (300 mA) was set. The time was set according to molecular weight. The membranes were placed in 5% skim milk and left at room temperature for 2 h. The corresponding antibody was incubated at 4 °C overnight. The secondary antibody corresponding to these membranes was applied on day 2. The results were scanned by the Odyssey Infrared Imaging System (LI-COR). Primary antibodies against the following antigens were used: T-PA (1:1000, Proteintech, Chicago, IL, USA), PKM2 (1:2000, Proteintech, Chicago, IL, USA), GAPDH (1:1000, Proteintech, Chicago, IL, USA).

### 4.14. Quantitative Real-Time Polymerase Chain Reaction (qRT-PCR)

Cells or tissues were lysed with TRIzol reagent (Invitrogen, Carlsbad, USA) to obtain the total RNA. Then, chloroform was added, supernatant was collected after centrifugation, isopropyl alcohol was added, and centrifugation was allowed. After the precipitation was collected and centrifuged with 75% ethanol, the RNA was obtained by adding the precipitation to DEPC water (Biosharp, Hefei, China). The RNA concentration was measured by NanoDrop ND-8000 (Thermo Fisher Scientific, Waltham, MA, USA). RNA was transcribed into cDNA using reverse transcription kits (TOYOBO, Tokyo, Japan) and reverse transcription applications (BIO-RAD). A quantitative real-time polymerase chain reaction was performed using SYBR (Roche) and primer (Invitrogen, Carlsbad, USA).

Primers:

t-PA-Forward: AAGGAGGGACAGGAATGCTT

t-PA-Reverse: CACACCTGTCCACAGTCCAC

PKM2-Forward: GCCGCCTGGACATTGACTC

PKM2-Reverse: CCATGAGAGAAATTCAGCCGAG

GAPDH-Forward: AGGTCGGTGTGAACGGATTTG 

GAPDH-Reverse: TGTAGACCATGTAGTTGAGGTCA

### 4.15. Single-Cell RNA Sequencing (Sc-RNA)

The single-cell-RNA-sequencing dataset (GSE221978) was downloaded from the Gene Expression Omnibus (GEO). The Sc-RNA-sequencing data were processed using Seurat version 5.0.1. Quality control was performed and cells with more than 10% mitochondrial genes were filtered out. The tSNE map of the sham group and DVT group was constructed with FindClusters, with a resolution of 0.5. The top-five different-expressed genes among different clusters with highest log2 fold change were used to perform cell annotation. Cell annotation was performed using cell marker 2.0. The expression of the PKM2 genes in different cell types in the sham group and DVT group was displayed using FeaturePlot and DotPlot functions. A Wilcoxon rank–sum test was used to find different-expressed genes between the sham and DVT group. For the different-expressed gene identify criteria, genes with adjusted *p* < 0.05 and absolute log2 fold change > 1 were regarded as different-expressed genes. The Gene Ontology (GO) dataset was used to undergo the pathway enrichment analysis about biological process based on the up-regulated different-expressed genes of endothelial cells in the DVT group. Data analysis was carried out in version R 4.3.0.

### 4.16. Data Analysis

The experimental data and representation methods of this experiment were mean ± SEM. Student’s *t*-test was used for comparison between two groups, and a one-way analysis of variance (ANOVA) was used for comparison between three or more groups. GraphPadPrism 9.5.2 software was used for statistical analysis and plotting of the obtained data, and *p* < 0.05 was used to define the difference as statistically significant.

## 5. Conclusions

In this study, our findings suggest that intramuscular administration of BBR influences the arterial fibrinolytic system, potentially by modulating t-PA activity. Moreover, BBR appears to regulate PKM2 expression, which in turn affects t-PA expression and fibrinolysis. While these results are promising, further research is needed to elucidate the underlying mechanisms and assess the therapeutic potential of BBR for thromboembolic diseases in more extensive clinical investigations.

## Data Availability

The original contributions presented in the study are included in the article/Supplementary Materials, further inquiries can be directed to the corresponding author.

## Supplementary Materials

The following supporting information can be downloaded at: https://www.mdpi.com/article/10.3390/ph17091219/s1, Figure S1: Dose of BBR in vivo and vitro experiments; Figure S2: Intramuscular injection of BBR prolongs clot time; Figure S3: BBR regulates PKM2 and the enrichment process.
