# Supplementary material for: Berberine Targets PKM2 to Activate the t-PA-Induced Fibrinolytic System and Improves Thrombosis
Source: Pharmaceuticals (Basel). 2024 Sep 17;17(9):1219. doi: 10.3390/ph17091219 (PMC11434879; doi:10.3390/ph17091219)
Supplement: Supplementary file 1 [file pharmaceuticals-17-01219-s001.zip › pharmaceuticals-3149067-supplementary.pdf]

Supplementary Figure S1

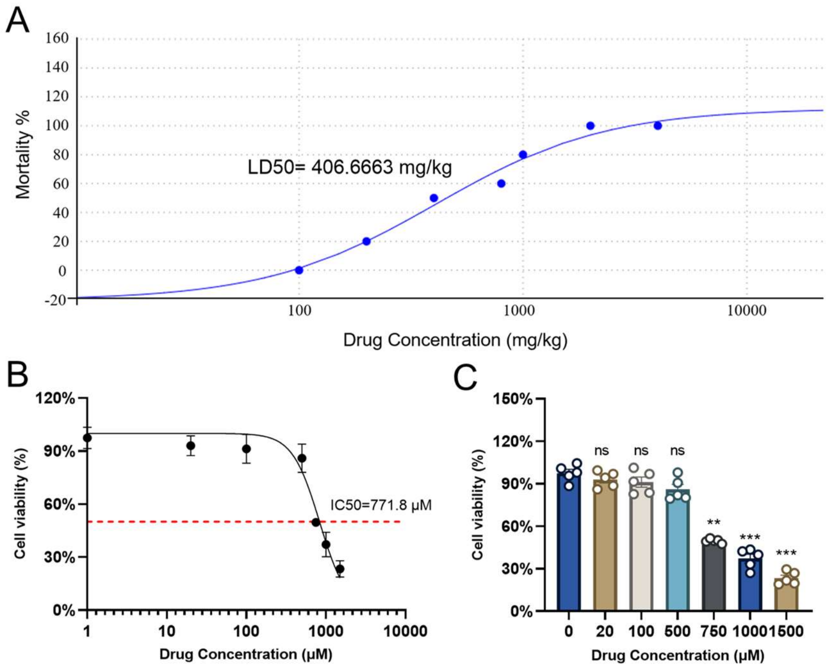

Supplementary Figure S1. Dose of BBR in vivo and vitro experiments. (A) Rats were injected intramuscularly BBR with seven concentrations 100, 200, 400, 800, 1000, 2000, and 4000 mg/kg. The calculate of LD50 is 406.6633 mg/kg. n=10. (B) HUVECs were treated with BBR with five concentrations 0, 20, 100, 500, 750, 1000 and 1500 μM. The calculate of IC50 is 731.1 μM. n=5. (C) The cell viability in five groups with differemt concentrations. **P < 0.01, ***P < 0.001 vs 0 μM. Data were expressed by mean ± SEM.

Supplementary Figure S2

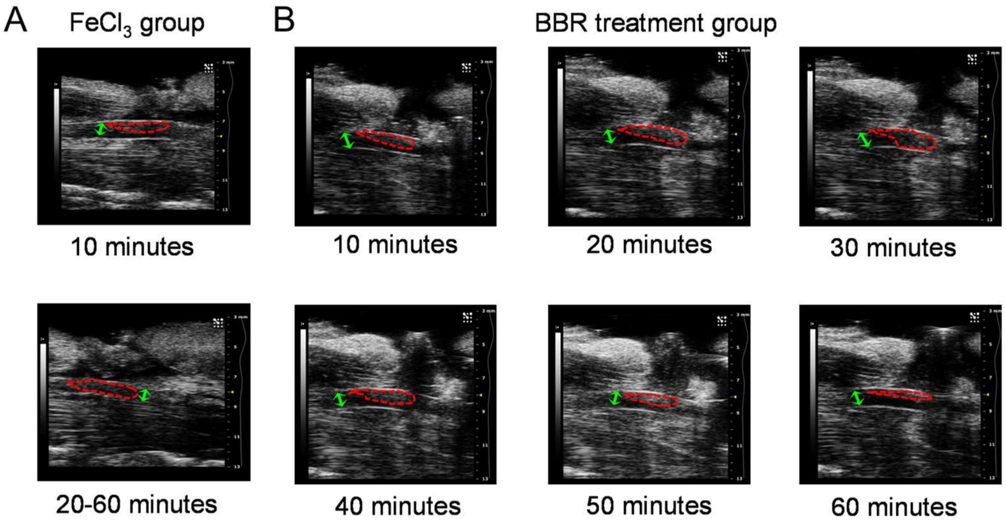

Supplementary Figure S2. Intramuscular injection of BBR prolongs clot time. (A) Doppler ultrasonography for detecting carotid thrombosis in $FeCl_3$ group. The complete blockage time

was 20 minutes. n=5. (B) Doppler ultrasonography for detecting carotid thrombosis in BBR treatment group. The complete blockage time was 30 minutes. n=5. $FeCl_3$ group and BBR treatment group were monitored for thrombus status from the start of treatment until 60 minutes. The red circles are thrombus and green arrowheads showed arterial width.

Supplementary Figure S3

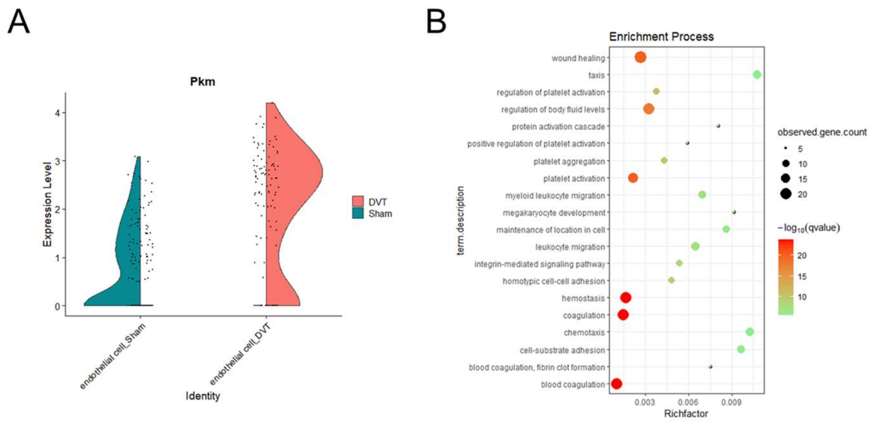

Supplementary Figure S3. BBR regulates PKM2 and the enrichment process. (A) Violin Plot of PKM2 gene expression in all the cell types in DVT (Left) and sham groups (Right). (B) GO enrichment analysis in DVT and sham groups.
